# Sensitive detection and measurement of oligogalacturonides in *Arabidopsis*

**DOI:** 10.3389/fpls.2015.00258

**Published:** 2015-04-21

**Authors:** Daniela Pontiggia, Jacopo Ciarcianelli, Gianni Salvi, Felice Cervone, Giulia De Lorenzo, Benedetta Mattei

**Affiliations:** Laboratory of Plant Physiology, Department of Biology and Biotechnology “C. Darwin”, Istituto Pasteur – Fondazione Cenci Bolognetti, Sapienza University of RomeRome, Italy

**Keywords:** plant cell wall, *Arabidopsis thaliana*, oligogalacturonides, pectin, HPAEC-PAD, MALDI-TOF-MS, LC-MS/MS

## Abstract

Oligogalacturonides (OGs) are pectin fragments derived from the partial hydrolysis of the plant cell wall pectin; they are elicitors of various defense responses. While their activity is well documented, the detection of OGs produced *in planta* is still a challenging task. A protocol has been developed for the extraction and analysis of OGs from small samples of *Arabidopsis* tissues by using fluorescent labeled OGs, which allowed to monitor the efficiency of extraction. An efficient recovery was obtained by using a combination of calcium chelating agents at acidic pH. Off-line coupling of high performance anion exchange chromatography with matrix assisted laser desorption ionization- time of flight-mass spectrometryor nanoESI-Orbitrap-MS/MS was used for the identification and characterization of oligosaccharides. The protocol was successfully applied to detect OGs by using low amounts (50 mg) of *Arabidopsis* leaves and very low amounts (30 mg) of senescent leaves. The protocol was also successfully used to detect OGs in *Arabidopsis* cell wall material digested with pectinases. The proposed extraction protocol followed by sensitive and high-resolution analysis methods allowed detection of OGs released from the cell wall in *Arabidopsis* tissues by using minimal sample material. The protocol may be useful to study OG-triggered plant immunity and cell wall remodeling during *Arabidopsis* growth and development.

## Introduction

The primary cell wall of plants is mainly composed of polysaccharides (pectins, hemicelluloses, cellulose) that are assembled into interconnected networks. The structure of the cell wall not only determines the mechanical properties of the plant tissue but also plays an active role in many aspects of plant development and plant–pathogen interactions. Pectin, one of the main components of the plant cell wall, is the most complex polymer in the wall, comprising a large number of different monosaccharides and linkages (Senechal et al., 2014). The main component of pectin is homogalacturonan (HG), a homopolymer of (1–4)-α-D-galacturonic acid (GalA) units (Carpita and Gibeaut, 1993). HG is synthesized in a methylated form in the Golgi apparatus and is secreted to the cell wall, where it is de-esterified *in muro* by pectin methyl esterases (PMEs). HG blocks of de-esterified residues form the so-called “egg-box” structures by calcium cross-bridges between chains (Cabrera et al., 2008; Caffall and Mohnen, 2009).

During infection, phytopathogenic microbes degrade HG by the action of endo-polygalacturonases (PGs) and other pectic enzymes (Cervone et al., 1989). In the cell wall, the interaction between PGs and specific plant inhibitors (polygalacturonase-inhibiting proteins: PGIPs) favors the formation of Oligogalacturonides (OGs) with degree of polymerization (DP) from 10 to 15 that function as signals for the activation of the plant innate immunity (Mattei et al., 2005). Under physiological conditions, OGs are thought to be released from the plant cell wall by the action of endogenous wall-remodeling pectic enzymes activated during growth and development or induced by mechanical damage (Orozco-Cardenas and Ryan, 1999).

Oligogalacturonides are regarded as Damage-Associated Molecular Patterns (DAMPs) and, during infection and wounding, may elicit a wide range of defense responses, including accumulation of phytoalexins (Davis and Currier, 1986), glucanase, and chitinase, deposition of callose, production of reactive oxygen species and nitric oxide (Ferrari et al., 2013). Despite the numerous studies demonstrating their involvement in defense, their presence and detection *in planta* has been so far elusive and remains challenging. Only in the presence of massive tissue maceration, which occurs at the later stages of infections, OGs have been detected in tomato and strawberry fruit tissues (An et al., 2005; Osorio et al., 2008).

The recovery of sufficient amounts of OGs for analytical studies is difficult, due to the presence of ionic interactions and cross bridges between pectin chains, and additional degradation or modification problems. We have developed a procedure for the extraction of OGs from plant cell walls of *Arabidopsis* that allows their detection in minimal amount of plant tissue or of wall material subjected to treatment with pectic enzymes, as required in oligo mass profiling (OLIMP) of cell wall polysaccharides (Günl et al., 2010). The procedure can be useful for the analysis of OGs *in planta* during early stages of pathogenesis or during plant development.

## Materials and Methods

### Chemicals and Reagents

Absolute ethanol, acetone, chloroform, dimethyl sulfoxide (DMSO), methanol, glacial acetic acid, tri-fluoroacetic acid (TFA) are ACS grade solvents and were purchased from Carlo Erba reagents (Rodano, Milano, Italy). Ultrapure water was purified by a Milli-Q water purification system (Millipore, Milford, MA, USA). 6-aminofluorescein (6A), ammonium formate, 1,2-diaminocyclohexanetetraacetic acid (CDTA), ammonium acetate, dihydroxybenzoic acid (DHB), sodium cyanoborohydride (NaCNBH_3_), ammonium oxalate were purchased by Sigma–Aldrich (St. Louis, MO, USA).

#### Reagent Setup

Chelating solution 1 (ChA1): 50 mM CDTA dissolved in 1 M NaOH.

Chelating solution 2 (ChA2): 50 mM CDTA, 50 mM ammonium oxalate, 50 mM ammonium acetate, pH 5.5 in ultrapure water.

Growth medium for *Arabidopsis*: 4.4 gl^-1^ Murashige and Skoog basal medium containing 1% sucrose and 0.8% agar in ultrapure water.

### Preparation of Elicitor-Active of Oligogalacturonides (OGs)

Oligogalacturonides were prepared according to the protocol described in Bellincampi et al. (1993). High molecular weight un-methylated polygalacturonic acid (PGA; Alfa Aesar) was solubilized in 100 ml of sodium acetate 50 mM, pH 5.0 to a concentration of 2% (w/v). The solution was digested for 180 min with 0.018 RGU of A. niger endoPG (kind gift from the laboratory of Prof. Jaap Visser, Wageningen University), and the enzyme was inactivated by boiling the digest at 100∘C for 10 min in a water bath. After enzyme inactivation, the sample was diluted with 50 mM sodium acetate to a concentration of 0.5% PGA. To precipitate OGs, ethanol was added to the digest to a final concentration of 17% (v/v); the sample was incubated overnight at 4∘C with shaking and then centrifuged 30 min at 30000 g. OGs, recovered in the pellet, were re-dissolved in water, dialyzed against water in a dialysis tube with a molecular mass cut off (MWCO) of 1000 Da (Spectra/Por®;), and lyophilized.

### Labeling of Oligogalacturonides

Oligogalacturonides were labeled according to a previously described protocol (Grun et al., 2006). Twenty μmoles of 6-aminofluorescein were dissolved in 70 μl of DMSO by vigorous mixing and heating at 65∘C for 1 min. Thirty microliter of glacial acetic acid were added to the solution followed by 100 μmoles of NaCNBH_3_, which were dissolved completely after heating at 65∘C for about 1 min. Next, the reaction mixture (10 μl) was added to a solution of 0.1 mg OGs dissolved in 10 μl ultrapure water. The labeling reaction was carried out at 65∘C for 3 h and labeled OGs were precipitated in absolute ethanol. The pellet was recovered by centrifugation and washed twice with 80% ethanol. Labeled OGs were dissolved in ultrapure water and dialyzed against water using 1000 Da MWCO tubes. After dialysis, samples were vacuum dried.

### Polyacrylamide Gel Electrophoresis

**FIGURE 1:**
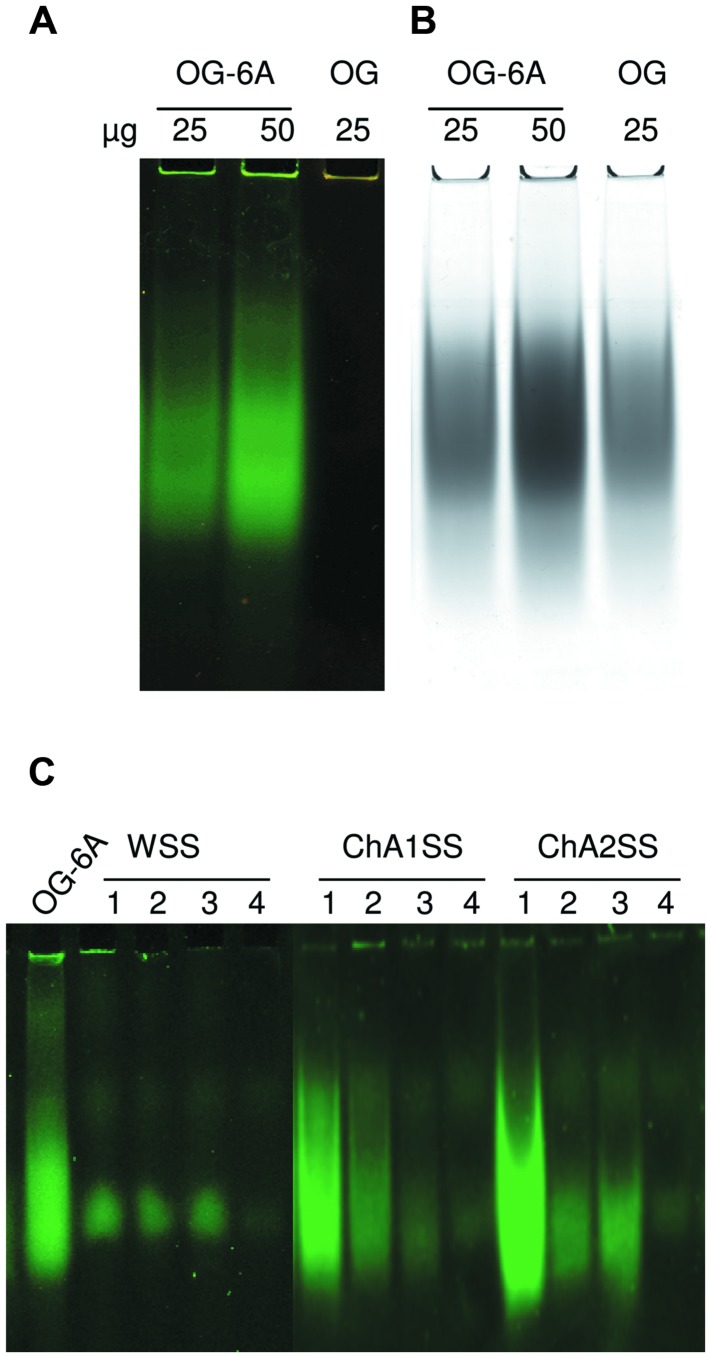
**Analysis of labeled Oligogalacturonides (OGs) by non-denaturing polyacrylamide gel electrophoresis**. **(A)** Labeled (OG-6A, 25, and 50 μg) and unlabeled OGs (OG, 25 μg). Image was acquired using Typhoon 9200 laser scanner (λ = 520 nm). **(B)** The same gel was stained with Ruthenium Red. **(C)** Labeled OGs extracted from leaf tissues of *Arabidopsis*. Fractions WSS, ChA1SS e ChA2SS (obtained by sequential extraction of alcohol insoluble solids (AIS) from the different samples) were subjected to non-denaturing polyacrylamide gel electrophoresis. Image was acquired using Typhoon 9200 laser scanner (λ = 520 nm). Samples: (1) leaves infiltrated with 125 μg labeled OGs (OG-6A); (2) leaves infiltrated with 25 μg OG-6A; (3) not infiltrated leaves to which 10 μg OG-6A were added after tissue homogenization; (4) leaves infiltrated with H_2_O. OG-6A (20 μg) were loaded as positive controls.

Labeled OGs (25 and 50 μg) or unlabeled OGs (25 μg) were added to loading buffer solution (TBE 1X, 4% glycerol, 0.1% bromophenol blue). The samples were loaded on a polyacrylamide gel (15% acrylamide-bis acrylamide 29:1, TBE 20X, 0.1% APS, 0.16% TEMED) and run at constant voltage of 100 V for 20 min. Gel images were acquired using a Typhoon 9200 laser scanner (Ge-Healthcare) and the following scan settings: excitation wavelength of 480 nm and emission wavelength of 520 nm. Next, the gel was stained with Ruthenium Red (0.02%, w/v) and image was acquired with a GelDoc (BIO-RAD) (**Figures 1A,B**).

### MALDI-TOF (Matrix Assisted Laser Desorption Ionization- Time of Flight) Mass Spectrometry

Ten microliter of sample (standard OGs, WSS or ChASS, or fractions) dissolved in water were pre-treated for 10 min with BioRex MSZ 501 cation exchange resin beads (BIO-RAD). Sample (1 μl) was added to 1 μl of 5% DHB in 0.2% TFA and spotted onto the MALDI plate (Bioscience). The spots were placed to quickly crystallize under vacuum and analyzed by matrix assisted laser desorption ionization- time of flight (MALDI-TOF) mass spectrometry (MS) with a Voyager DE-STR instrument (Applied Biosystems, Beverly, MA,USA).

### Plant Material and Growth Conditions

*Arabidopsis thaliana* Columbia-0 plants were grown in a growth chamber maintained at 22∘C and 70% relative humidity, with a 12-h photoperiod. Etiolated seedlings were grown by germinating seeds, after 2-day stratification at 4∘C, on plates containing 20 ml of growing medium (4.4 gl^-1^ Murashige and Skoog basal medium containing 1% sucrose and 0.8% agar dissolved in ultrapure water). Plates were placed in growth chamber at 22∘C with light for 2 h and then wrapped in aluminum foil for 5 days.

### Preparation of AIS (Alcohol Insoluble Solids)

Two leaves (about 50 mg of fresh weight) of 4-week-old plants were infiltrated each by using a syringe without the needle, with sterile ultrapure water or 250 μl of a solution containing labeled OGs (100 ng/μl or 500 ng/μl). Leaves were immediately excised, frozen in liquid nitrogen and homogenized for 2 min at 30 Hz in a mixer mill MM301 (RETSCH), using inox beads (6 mm diameter). Ground tissues were re-suspended in 1 ml 70% ethanol, centrifuged 10 min at 12000 *g*. The pellet was washed twice with a chloroform:methanol (1:1, vol/vol) mixture, vortexed, and centrifuged at 14000 *g* for 10 min. The pellet was washed twice with acetone and centrifuged at 14000 *g* for 10 min. Washing was repeated twice, until disappearance of chlorophyll. The pellet, containing the alcohol insoluble solids (AIS) was dried at room temperature under a chemical hood overnight.

### Extraction of the Water Soluble Solid (WSS) Fraction

Alcohol insoluble solids fraction was re-suspended in 100 μl ultrapure water and kept overnight at 4∘C with shaking. After centrifugation for 20 min at 18000 *g*, the water soluble solid (WSS) fraction was recovered as the supernatant. WSS fraction was analyzed by polyacrylamide gel electrophoresis or MS. The pellet, indicated as Residue 1, was recovered for further extraction.

### Extraction of the Chelating Agent Soluble Solid (ChASS) Fractions

Residue 1 fraction was re-suspended in 100 μl chelating agent solution 1 (ChA1) and incubated overnight at 4∘C. After centrifugation for 20 min at 18000 *g*, the supernatant containing the chelating agent 1 soluble solid (ChA1SS) fraction was recovered. The pellet (Residue 2) was re-suspended in 100 μl chelating solution 2 (ChA2) and after centrifugation for 20 min at 18000 *g*, the supernatant containing the chelating agent 2 soluble solid (ChA2SS) fraction was recovered. Polysaccharides contained in the ChASS fractions were precipitated with either 80% ethanol at -20∘C overnight or sequentially precipitated with 20 and 80% ethanol at -20∘C overnight, as specified.

**FIGURE 2:**
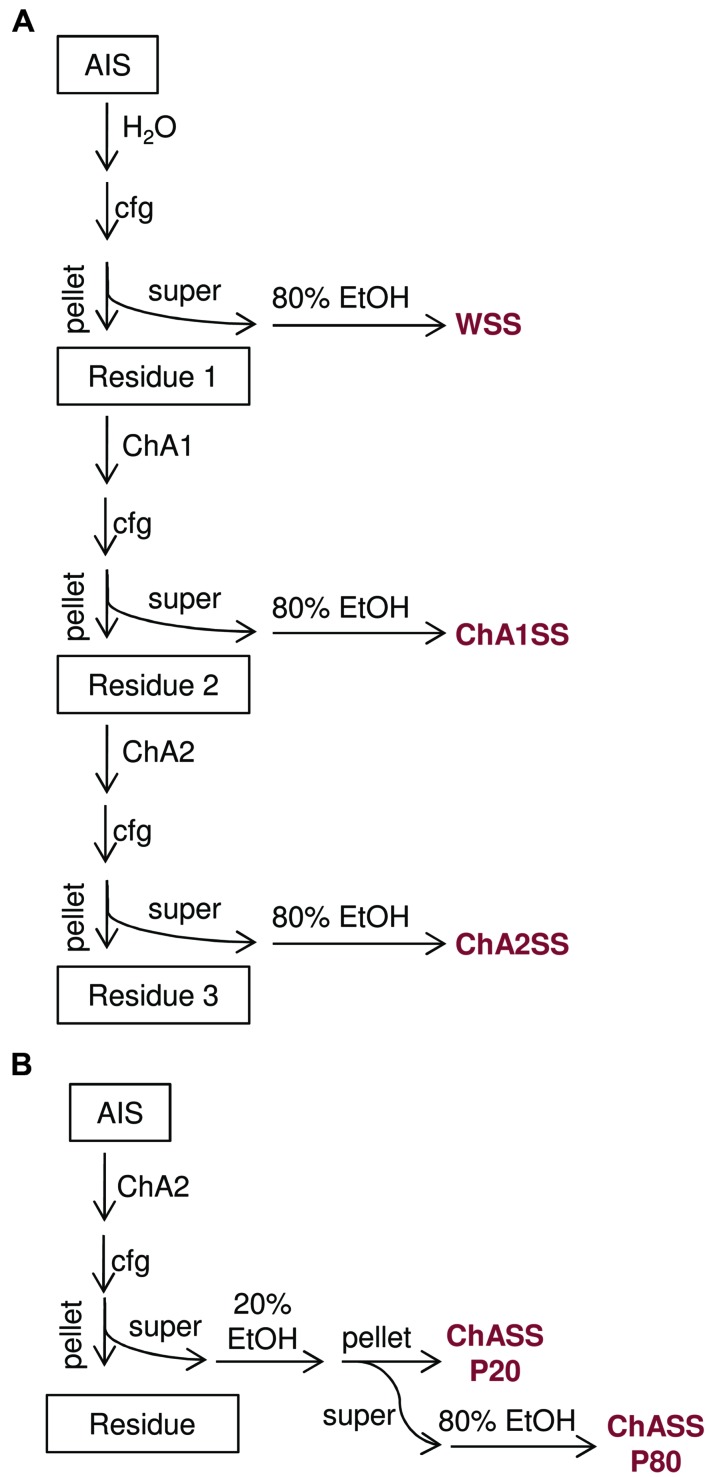
**Flow charts of the procedures for extraction of oligogalacturonides from AIS**. **(A)** The sequential extraction procedure, AIS is fractionated into water soluble solids (WSS), chelating agent 1 soluble solids (ChA1SS), and chelating agent 2 soluble solids (ChA2SS), which are subsequently recovered by ethanol precipitation. **(B)** The simplified extraction procedure, AIS is subjected to a single extraction step using ChA2 buffer. The resulting fraction (ChASS) is then fractionated using two sequential precipitation steps with 20% ethanol and 80% ethanol, to obtain the ChASS P20 and ChASS P80 precipitates, respectively.

Pellets obtained by centrifugation at 18000 *g* for 10 min were washed twice with 80% ethanol, and dried 10 min under a flow hood. Polysaccharides were dissolved in 100 μl ultrapure water (**Figure 2A**).

### Quantitative Evaluation of OG Recovery

Fluorescent OGs were either infiltrated into (25 μg/50 mg of plant tissue) or added to leaves of 5-week-old *Arabidopsis* plants, and recovered using the extraction protocol illustrated in **Figure 2B**. After extraction, fluorescence in the ChASS was measured by Fluorometer GlowMax (Promega, France) using the blue optical kit with an excitation λ = 490 nm and an emission λ = 510–570 nm. In a parallel experiment, different amounts of unlabeled OGs (10, 25, 62.5, 125 μg per 50 mg of plant tissue) were infiltrated into leaves, and recovered using the same extraction protocol. In this case, the percentage of OG recovery was measured by High performance anion exchange chromatography with pulsed amperometric detection (HPAEC-PAD) analysis.

### Oligo Mass Profiling (OLIMP)

For the profiling of HG, the AIS was digested for 17 h at 37∘C in a solution of 50 mM ammonium formate, pH4.5, containing one unit of PME from *Aspergillus aculeatus* (Novoshape, Novozymes, Denmark) and 1 unit of endo-PG from *Aspergillus niger* (Megazyme). Fragments were extracted using 100 μl ChA2 solution, precipitated with absolute ethanol at -20∘ C overnight, recovered in the pellet after centrifugation at 18000 *g* for 10 min and washed twice with 80% ethanol.

### NanoESI MS–MS

Oligogalacturonides were resuspended in ultrapure water and treated for 10 min with BioRex MSZ 501 cation exchange resin beads (BIO-RAD) at room temperature. The OG solution was mixed with methanol in 80:20 (v/v) ratios and analyzed by off-line static nano-ESI LTQ-Orbitrap mass spectrometer (Thermo). Static injection was obtained with a coated PicoTip emitter (OD/ID 1.2 / 0.69, tip 2 μm, New Objective) filled with the sample and placed at the nanoESI source inlet, using a capillary voltage of 4.2 kV and capillary temperature of 200∘C.

### High Performance Anion Exchange Chromatography with Pulsed Amperometric Detection (HPAEC-PAD)

Thirty μl of sample (OGs or aliquots of the ChA2SS fractions) were analyzed by high-performance anion-exchange chromatography with pulsed amperometric detection (HPAEC-PAD) using a 3 × 250 mm anionic exchange column Carbopac PA200 with guard column (Dionex Thermo Fischer) mounted on a ICS3000 (Dionex Thermo Fischer) HPLC.

Before injecting the samples, the column was equilibrated for 10 min with a flow of 90% Eluent A (50 mM potassium hydroxide) and 10% Eluent B (50 mM potassium hydroxide, 1 M potassium acetate). Separation was obtained at a flow-rate of 0.3 ml/min applying a linear gradient from 90 to 20% of eluent A and from 10 to 80% of eluent B for 35 min. Finally, the column was washed with 100% eluent B. Pectic fragments were detected by a pulsed amperometric detector using a gold electrode with the waveform A, according to the manufacturer instructions.

## Results and Discussion

### Extraction Protocol

**FIGURE 3:**
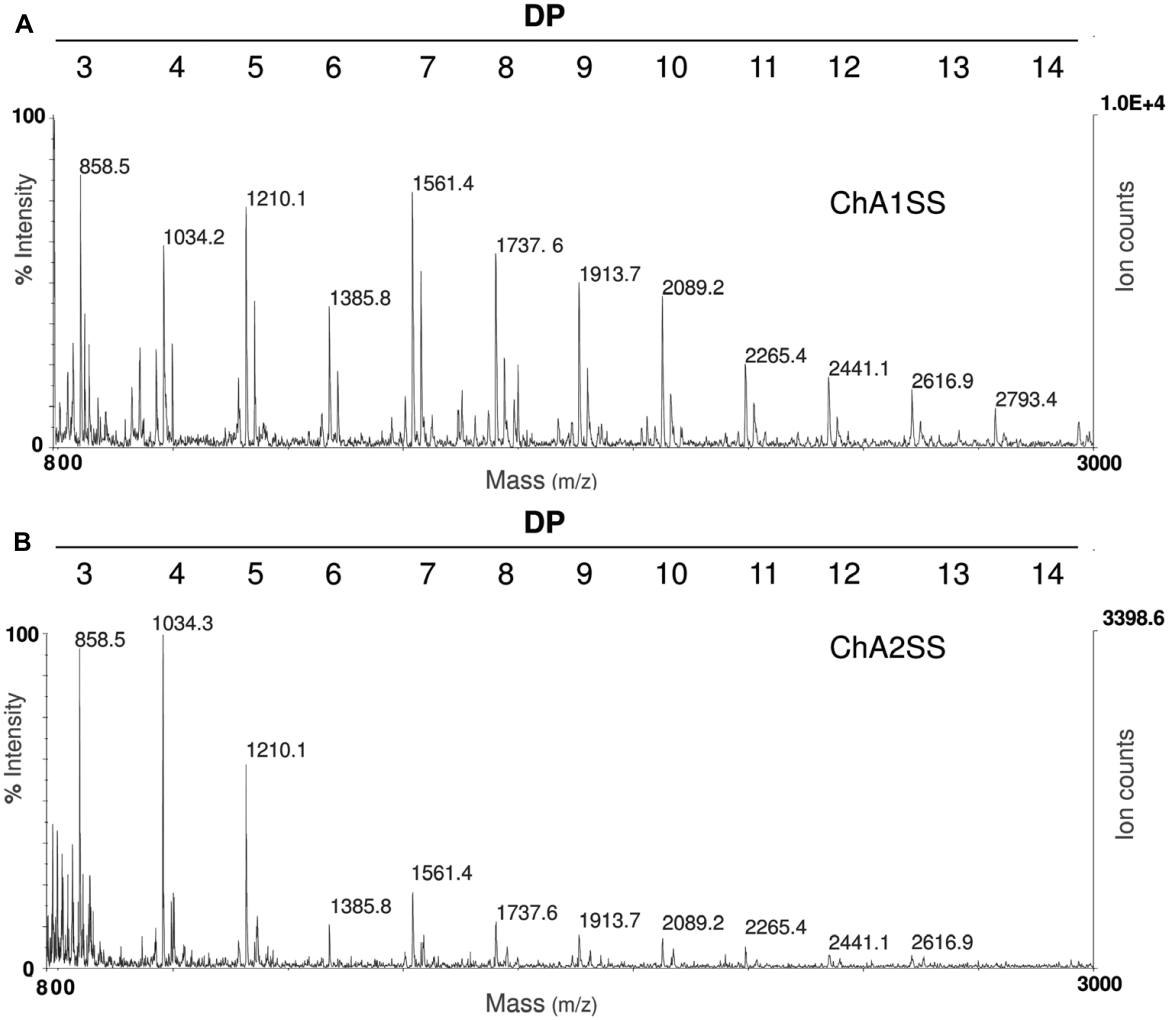
**Matrix assisted laser desorption ionization- time of flight (MALDI-TOF) mass spectrometry (MS) analysis of OGs recovered in the fractions ChA1SS **(A)** and ChA2SS **(B)** showing a different distribution of degree of polymerization (DP): the ChA1SS fraction shows a distribution of DP between 3 and 14 with the most intense peak corresponding to DP 7, while in the ChA2SS fraction the most intense peak corresponds to DP 4**.

In order to set up the extraction protocol and assess the efficiency of recovery, OGs were labeled *in vitro* to be subsequently infiltrated into *Arabidopsis* leaves. OGs with DP between 5 and 26, were prepared and labeled with 6-aminofluorescein (6A) as described in Materials and Methods. Labeling with 6A allowed easy visualization of the labeled oligosaccharides after electrophoresis on polyacrylamide gel (**Figures 1A,B**). The labeled OGs (OG-6A) were analyzed by MALDI-TOF MS to evaluate the quality and quantity of the products obtained and to verify that the distribution of molecular weights had not been altered by the labeling and extraction procedure. After syringe infiltration of labeled OGs, leaves (about 50 mg) were immediately frozen for extraction of de-proteinized total cell walls (AIS: Alcohol-Insoluble Solids). In an additional sample, OG-6A (10 μg) were added to frozen leaves immediately after homogenization. AIS was sequentially fractionated into water-soluble solids (WSS), Chelating Agent 1 (CDTA/NaOH)-Soluble Solids (ChA1SS) and Chelating Agent 2 (CDTA/NH_4_^+^-oxalate/NH_4_^+^-acetate)-Soluble Solids (ChA2SS), which were recovered by 80% ethanol precipitation (**Figure 2A**). Electrophoresis on polyacrylamide gel detected only a very small amount of OG-6A in the WSS fractions, also in those samples in which OG-6A had been added after homogenization of the tissue (**Figure 1C**), indicating that OGs are likely retained by the cell wall matrix through ionic interaction. OG-6A were instead abundant in the two fractions sequentially extracted with chelating agents ChA1 and ChA2 (**Figure 1C**). It is known that, whereas long and highly esterified pectin polymers can be readily extracted with cold and hot water, less esterified pectin requires extraction with divalent ion-sequestering agents such as oxalate or CDTA (Wang et al., 2002). Additional sequential extractions with 0.3 M NaCl, i.e., a high ionic strength solution that makes the galacturonan chains more flexible and extracts pectin held by steric interactions (Jarvis, 1982), or 1 M KOH, that extracts branched pectic polysaccharides and hemicellulose, were therefore performed. No OG-6A, however, were extracted (data not shown), suggesting that these oligosaccharides had been completely recovered in the fractions ChA1SS and ChA2SS. The DP of the recovered oligomers was analyzed by MALDI-TOF-MS (**Figure 3**). The two fractions display different distributions of OG size: the ChA1SS fraction shows a distribution of DP between 3 and 14 with the most intense peak corresponding to DP 7 (**Figure 3A**), while in the ChA2SS fraction the highest peak corresponds to DP 4 (**Figure 3B**).

**FIGURE 4:**
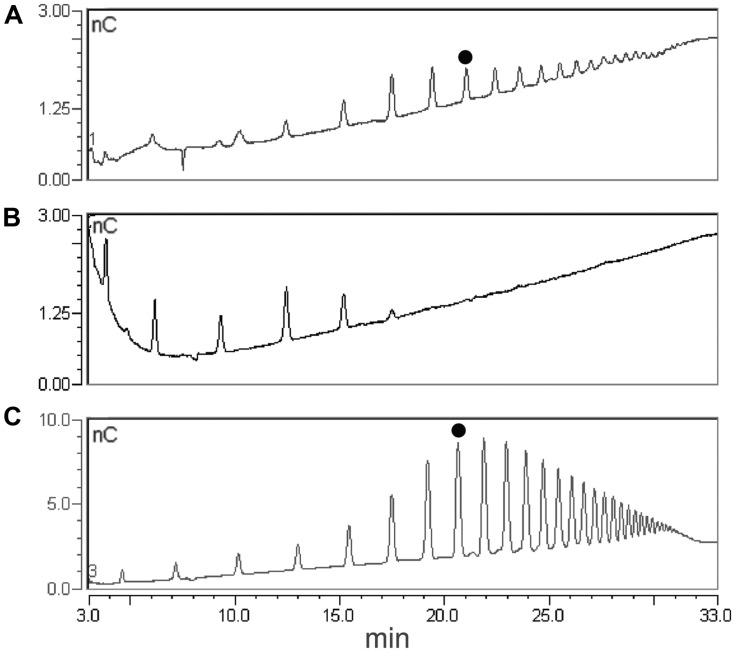
**High performance anion exchange chromatography with pulsed amperometric detection (HPAEC-PAD) profiles of fractions extracted from leaves using the simplified procedure**. **(A)** ChASS precipitated with 20% ethanol (ChASS P20). **(B)** Supernatant of ChASS in 20% ethanol in turn precipitated with 80% ethanol (ChASS P80). **(C)** Standard OGs with DP from 3 to 26. The peak corresponding to the oligomer with DP 10 is indicated with a black dot.

A simplified extraction procedure was designed on the basis of the results described above, including a single extraction step of the AIS with the ChA2 buffer, to obtain a ChA2SS fraction hereon indicated simply as ChASS, and a fractional ethanol precipitation (20% followed by 80% ethanol) to obtain the ChASS P20 and ChASS P80 precipitates, respectively, (**Figure 2B**). These precipitates were redissolved in water and subjected to HPAEC-PAD. This procedure was used to determine the presence of endogenous OGs, initially using 50 mg of leaf tissue from adult (3-week-old) *Arabidopsis* plants. HPAEC-PAD chromatograms show that oligomers with a distribution of DP between 8 and 20 were preferentially recovered in the pellet of the first precipitation (ChASS P20; **Figure 4A**), whereas shorter oligomers were enriched in the pellet of the 80% ethanol precipitation (ChASS P80; **Figure 4B**). The relative distribution of DP of the two fractions compared to the distribution of DP of the OGs before the infiltration (**Figure 4C**), shows a shift toward shorter oligomers, indicating that some degradation has occurred during the infiltration and extraction procedure, possibly due to the activity of endogenous PGs.

### Analysis of OGs in *Arabidopsis*

**FIGURE 5:**
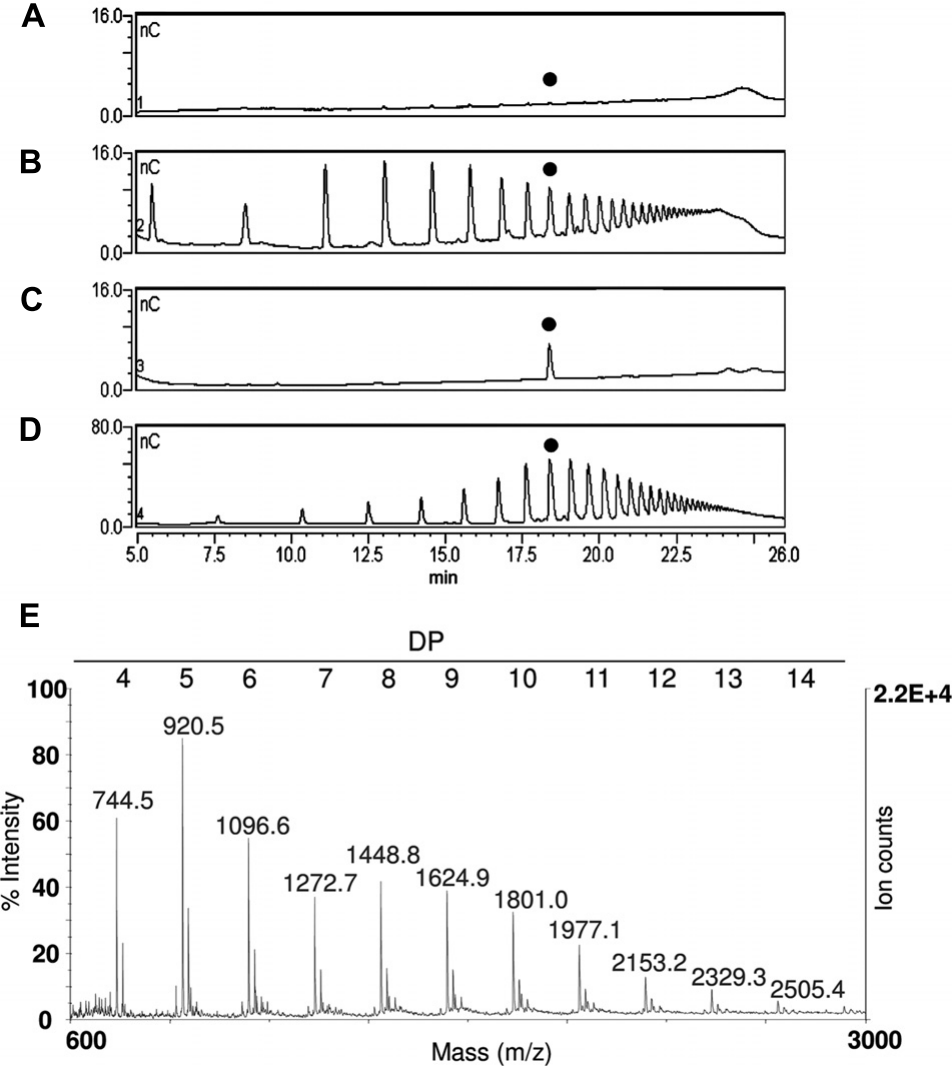
**The upper panel shows the HPAEC-PAD profiles of fractions extracted from leaves of plants of different age: **(A)** 3-week-old plants, **(B)** 7-week-old plants, **(C)** purified standard oligomer with DP 10, and **(D)** standard OGs with DP from 3 to 26**. The peak corresponding to the oligomer with DP 10 is indicated with a black dot. **(E)** MALDI-TOF mass spectrum of the oligomers extracted from 7-week-old plants.

The simplified extraction procedure described in the previous paragraph was then applied to amounts as low as 30 mg of leaf tissue from adult (3-week-old) and senescent (7-week-old) *Arabidopsis* plants. It is reported that senescence-related degradation of pectin may occur due to the up-regulation of genes encoding pectinesterase, pectate lyase, and polygalacturonase (Breeze et al., 2011). Indeed, while OGs were barely visible in the 3-week-old plants (**Figure 5A**), they were clearly detectable in the 7-week-old plants, mainly consisting of oligomers with a DP between 3 and 26 (**Figure 5B**), as compared to the standard OGs (**Figures 5C,D**). The recovered OGs from 7-week-old plants were analyzed by MALDI-TOF-MS, allowing a precise structural determination of the recovered oligomers (**Figure 5E**).

### Measurement of OG Recovery

**FIGURE 6:**
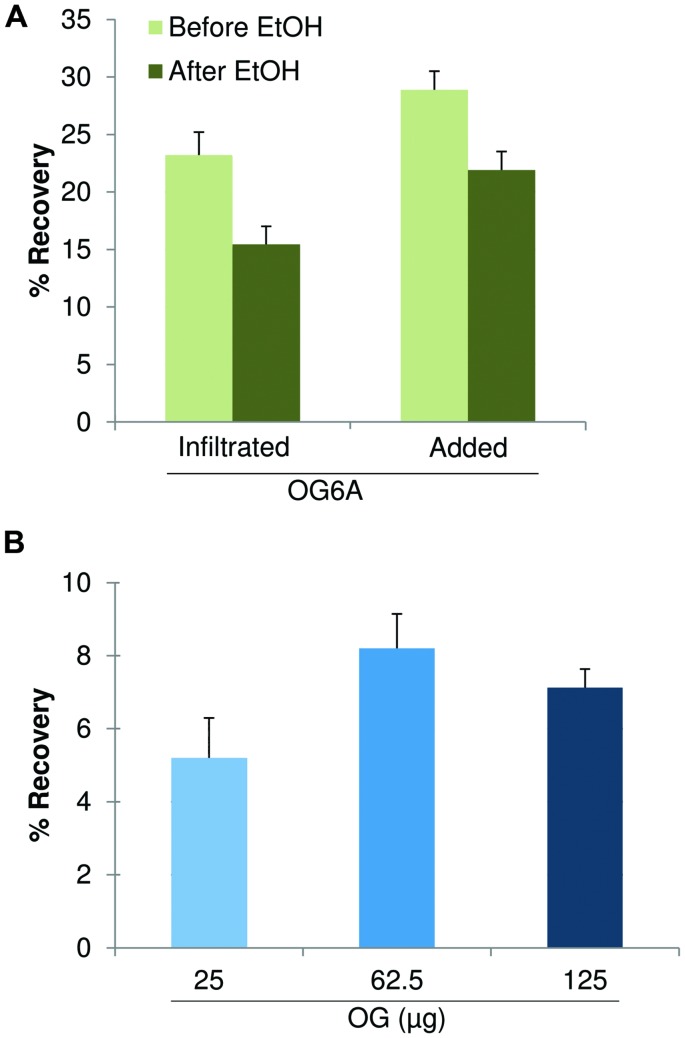
**Recovery of OGs**. **(A)** Recovery from 5-week-old leaves (50 mg) infiltrated with fluorescent labeled OGs (OG-6A, 25 μg at 100 ng/μl) or added with OG-6A (25 μg) before and after precipitation with 80% ethanol. **(B)** Recovery from leaf tissue (50 mg) infiltrated with 250 μl of unlabeled OGs at different concentrations (100, 250, and 500 ng/μl, corresponding to 25, 62.5, and 125 μg, respectively, of total OGs) after 80% ethanol precipitation. Each column represents the mean ± SE of three independent experiments.

The recovery of OGs was determined using two different quantitative approaches. In the first approach, a fixed amount of fluorescent labeled OGs (and H_2_O as a control) was infiltrated in 5-week old *Arabidopsis* leaves (25 μg/50 mg of leaves). The simplified extraction procedure was applied and fluorescence of the extracted fraction before and after precipitation with 80% ethanol was measured. Recovery was calculated by subtracting the fluorescence measured in the H_2_O-infiltrated sample and dividing the result by the fluorescence value obtained for the OG-infiltrated sample. The result was expressed as a percentage. The recovery was in the range 20–25% and about 15% before and after ethanol precipitation, respectively, (**Figure 6A**). In a parallel experiment fluorescent labeled OGs were added to leaves (25 μg/50 mg of leaves) and recovered using the same procedure. The recovery of added OGs was in the range 28–30% and about 20% before and after ethanol precipitation, respectively, (**Figure 6A**), suggesting that an important loss occurs during the procedure of AIS and ChASS preparation.

In the second approach, leaves were infiltrated with unlabeled OGs at different concentrations (40, 100, 250, and 500 μg/ml corresponding to 10, 25, 62.5, 125 μg per 50 mg of tissue). The simplified extraction procedure was applied and the extracted fraction, precipitated with 80% ethanol and redissolved in water, was analyzed by the HPAEC-PAD profiling method. The sum of the peak areas of oligomers with 9 ≤ DP ≤ 14 in the profile of recovered OGs was referred to the sum of the areas of the corresponding peaks in the profile of the OGs before infiltration. The recovery of OGs was in the range 5–9% (**Figure 6B**), except for the lower concentration (40 μg/ml), for which OG recovery was below the threshold of detection.

This value is lower than the recovery of labeled infiltrated OGs (around 15%), however, it must be considered that while the first method measures the fluorescence of the whole pool of infiltrated OGs, the HPAEC-PAD profile method measures only the fraction of oligomers with DP from 9 to 14. Since the profile of recovered OGs is different from the profile of OGs before infiltration (**Figure 4**) indicating that some degradation has occurred during the infiltration and extraction procedure, this may account for the difference in the recovery measured by the two methods.

In conclusion, these results show a relatively low recovery, likely due to loss of oligomers during the extraction procedure and the ethanol precipitation. However, the procedure allows the detection of OGs at 100 μg/ml, i.e., a concentration in the range of those used in experiments of immune response activation (Galletti et al., 2011).

### Analysis of OGs in Cell Wall Material upon Digestion with Pectinases (endoPG/PME)

**FIGURE 7:**
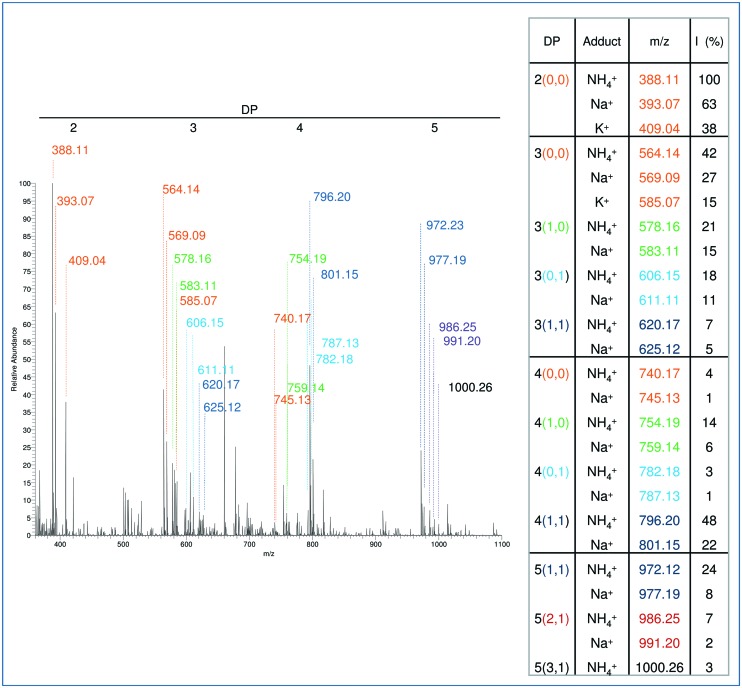
**Matrix assisted laser desorption ionization- time of flight mass spectrum of oligomers extracted after digestion of AIS with a mixture of PG and pectin methyl esterase (PME)**. The different oligomers detected in the MS spectrum are listed in the table shown on the right. For each of them, the DP is indicated as well as, in parentheses, the number of methyl and acetyl groups (for example, DP 4(1,1) indicates a tetramer carrying one methyl group and one acetyl group). The main ions detected by the analysis were assigned to Na^+^, K^+^, or NH4^+^ adducts on the basis of their m/z and literature data. Different colors in the table indicate different degrees of methylation or acetylation. For example, all the oligomers with one methyl group are indicated in green.

The characterization of plant cell walls with different susceptibility to microbial cell wall-degrading enzymes may help defining useful biochemical traits for selection of varieties with improved resistance to pathogens (Bellincampi et al., 2014) or better characteristics of bioconversion (Francocci et al., 2013). The analysis of the cell wall components performed by means of the oligosaccharide mass profiling (OLIMP) method has been proposed as a rapid tool for screening mutants with altered cell wall structure (Günl et al., 2010). It consists of a cell wall digestion using specific cell-wall degrading enzymes, followed by analysis of the released oligosaccharides by MALDI-TOF/MS. We tested whether our extraction procedure that uses chelating agents improves the detection of low-esterified pectic fragments obtained by OLIMP from small amounts of starting material. AIS (500 μg) from *Arabidopsis* seedlings was treated with 1 unit of PG and 1 unit of PME for 17 h at 37∘C. The simplified extraction protocol was then used to recover the fragments released by the enzymatic digestion. High resolution MS spectra of extracted fragments showed a complex pattern corresponding to different oligomers (**Figure 7**). The observed fragments in part correspond to those detected by OLIMP in a previous work (Lionetti et al., 2007). In addition, our procedure detected low-esterified oligomers, which are released by the action of PG/PME but are not easily extracted from the cell wall.

**FIGURE 8:**
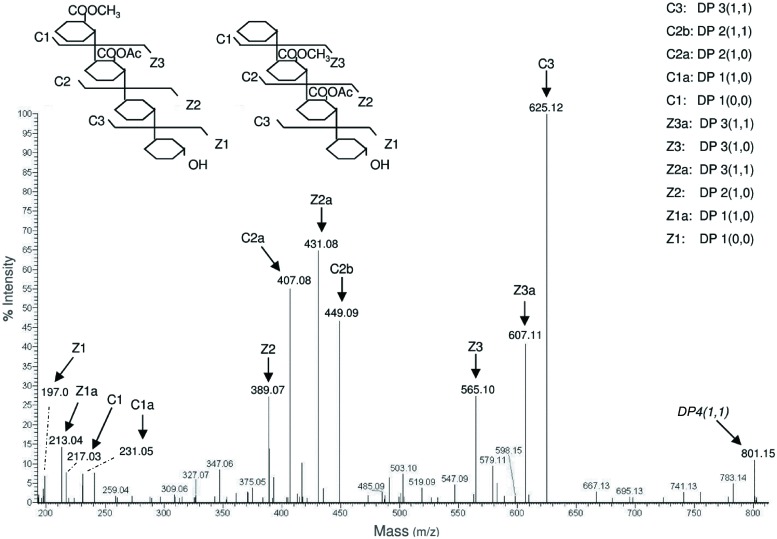
**MS/MS spectrum of the oligomer DP 4(1,1)**. This oligomer corresponds to a tetramer with m/z = 801.15 carrying one methyl group and one acetyl group. The spectrum shows the coexistence of two different structures, in which the methyl and acetyl groups are on different galacturonosyl residues. The nomenclature of fragment ions proposed by Domon and Costello (1988) was used.

The extracted samples were also suitable for MS/MS analysis using static nanoESI injection in an Orbitrap analyzer that allowed to determine two different structures corresponding to a single precursor mass (**Figure 8**). The position of the methyl and acetyl esters could be assigned on C1–C2 and C2–C3 [according to the nomenclature proposed by Domon and Costello (1988); see also Mutenda et al., 2007], indicating the presence of two isomeric structures that were not distinguishable on the basis of the MS spectrum alone. We concluded that our procedure is also useful for detection of the relative abundance of OGs with different structures and, coupled to the analysis by MS and MS/MS, can help elucidating the structure and accessibility of HG in different anatomical parts of *Arabidopsis* during growth and development.

## Conclusion

The procedure developed in this work can be used to characterize the OGs released *in planta* by endogenous enzymatic activity and to study the numerous dynamic processes that are associated with HG degradation and modification during defense and development. Very recently this procedure allowed to detect OGs generated *in planta* by a PGIP–PG fusion protein, designed to engineer *in vivo* the release of DAMPs (Benedetti et al., 2015). Moreover, the protocol allows the structural analyses of low-esterified pectic fragments that are released from the cell wall by exogenous enzymes in OLIMP analyses and are otherwise hardly detectable.

## Conflict of Interest Statement

The authors declare that the research was conducted in the absence of any commercial or financial relationships that could be construed as a potential conflict of interest.
